# Supplementary material for: PCSK9 stimulates Syk, PKCδ, and NF-κB, leading to atherosclerosis progression independently of LDL receptor
Source: Nat Commun. 2024 Mar 30;15:2789. doi: 10.1038/s41467-024-46336-2 (PMC10981688; doi:10.1038/s41467-024-46336-2)
Supplement: Supplementary file 1 — Supplementary Information [file 41467_2024_46336_MOESM1_ESM.pdf]

PCSK9 stimulates Syk, PKCδ, and NF-κB, leading to atherosclerosis progression independently of LDL receptor

Dasom Shin[1,2,3#], Soungchan Kim[1,2,3#], Hwan Lee[1,2,3#], Hyun-Chae Lee[1,2], Jaewon Lee[1,2], Hyun-woo Park[1,2,4], Mina Fukai[1,2,3], EunByule Choi[1,2,3], Subin Choi[1,2,3], Bon-Jun Koo[1,2,3], Ji-Hoon Yu[1,2,3], Gyurae No[1,2,3], Sungyoon Cho[1,2,4], Chan Woo Kim[5], Dohyun Han[2], Hyun-Duk Jang[1,2*], and Hyo-Soo Kim[1,3,4,6*]

[1]Center of CBT (Cell and BioTherapy), Seoul National University Hospital, Seoul, Republic of Korea

[2]Biomedical Research Institute, Seoul National University Hospital, Seoul, Republic of Korea

[3]Department of Molecular Medicine and Biopharmaceutical Sciences, Seoul National University, Seoul, Republic of Korea

[4]Program in Stem Cell Biology, Seoul National University College of Medicine, Seoul, Republic of Korea

[5]Department of Preclinical Trial, Laboratory Animal Center, Osong Medical Innovation Foundation (KBIO), Cheongju, Chungbuk, Republic of Korea

[6]Cardiovascular Center & Department of Internal Medicine, Seoul National University Hospital, Seoul, Republic of Korea

[#]These authors contributed equally to this work.

* These authors jointly supervised this work. and are co-corresponding authors.

Hyo-Soo Kim, MD, PhD

Department of Internal Medicine, Seoul National University Hospital, 101 Daehak-ro, Jongro-gu, Seoul 110-744, Republic of Korea

Tel: +82-2-2072-2226

Fax: +82-2-766-8904

E-mail: hyosoo@snu.ac.kr

Hyun-Duk Jang, PhD

Department of Biomedical Research Institute, Seoul National University Hospital, 101 Daehak-ro, Jongro-gu, Seoul 110-744, Republic of Korea

Tel: +82-2-2072-0218

E-mail: 65765@snuh.org

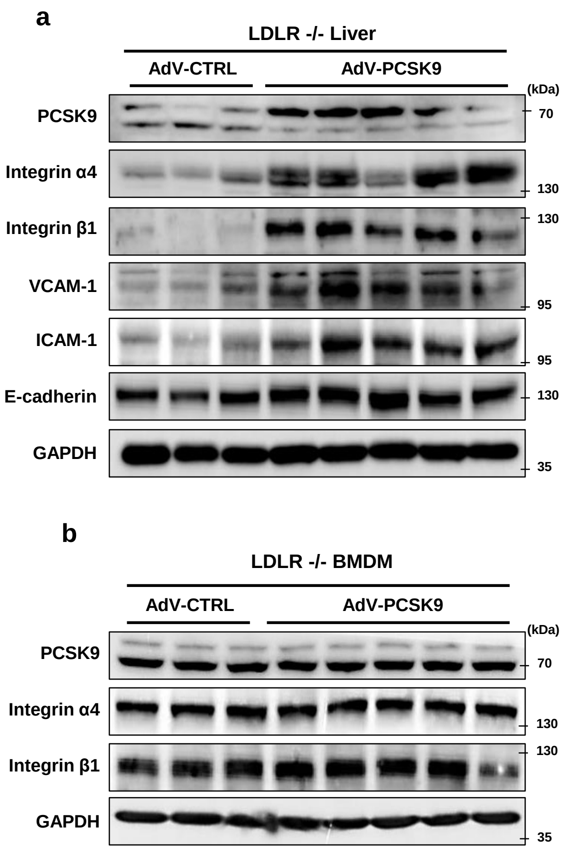

Supplementary Fig. 1

Expression of adhesion molecules in the animal experiment showing that PCSK9 aggravated atherosclerosis in *Ldlr*$^{-/-}$ mice (Figure 2). AdV-PCSK9 at 1×10^{11} infectious units/mouse was administered via the tail vein to mice on a high-fat diet. (a) Immunoblot analysis of the liver samples from *Ldlr*$^{-/-}$ mice showing that the expression of adhesion molecules (integrin-α4, integrin-β1, VCAM-1, and ICAM-1) along with PCSK9 was significantly higher in AdV-PCSK9 (N=5) mice than in AdV-CTRL (N=3) mice. (b) Immunoblot analysis of BMDMs from *Ldlr*$^{-/-}$ mice showed no significant changes in the expression of adhesion molecules (integrin-α4, integrin-β1).

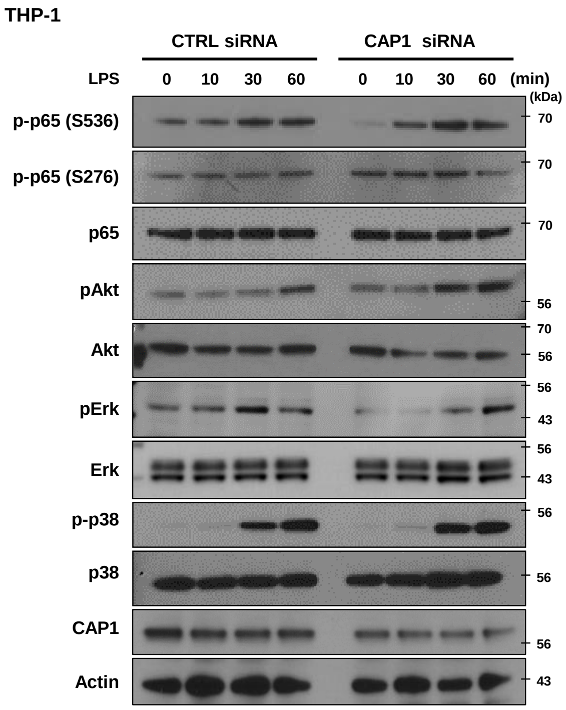

Supplementary Fig. 2

Effects of siRNA against CAP1 on LPS-induced phosphorylation of p65, Akt, Erk, and p38. THP-1 cells were transfected with CAP1 siRNA or non-targeting control (CTRL siRNA) and cultured for 3 days after LPS treatment in a time-dependent manner (0, 10, 30, and 60 min). Immunoblot analysis demonstrating that CAP1-deficient cells showed LPS-mediated pro-inflammatory signaling equivalent to control cells. The representative figures of three independent experiments are shown.

PCSK9 concentration and the level of phosphorylation in CAD patients

Patients	PCSK9 (serum)	pSyk	p-PKCδ	p-p65 (S276)	p-p65 (S536)
1	394.900	1.000	1.000	1.000	1.000
2	333.500	0.696	0.585	1.016	1.049
3	414.300	1.136	0.944	0.495	0.282
4	320.000	0.336	0.562	1.049	1.062
5	466.800	1.334	0.595	0.758	0.672
6	387.400	0.847	0.773	0.469	0.731
7	328.600	0.148	0.137	0.798	0.886
8	631.900	3.604	2.401	1.067	3.567
9	302.500	0.350	1.442	1.880	0.821
10	494.300	0.365	1.429	3.199	1.071
11	426.000	4.211	2.558	3.185	3.894
12	448.000	1.083	1.932	1.946	0.734
13	373.500	0.622	1.517	3.340	1.164
14	403.000	2.779	1.093	2.636	2.956
**Control**					
1	202.000	1.470	0.951	0.288	0.452
2	279.800	1.163	1.194	0.243	0.441
3	230.300	0.446	1.259	0.357	0.255
4	302.600	0.994	0.814	0.523	0.486
5	435.700	1.112	0.788	0.035	0.025

Supplementary Fig. 3

Correlation of serum PCSK9 and phosphorylation of Syk, PKCδ, and NF-κB in human PBMCs of CAD patients (N=14) and healthy donors (N=5) is described in Fig. 9a. The table shows the raw data of serum PCSK9 concentration (ng/mL) and quantified immunoblots of phosphorylated Syk, PKCδ, p65(S276), and p65(S536) in human PBMCs using ImageJ software (relative ratio). The quantified immunoblot raw data were normalized with patient data and plotted in a correlation graph against PCSK9 concentration.

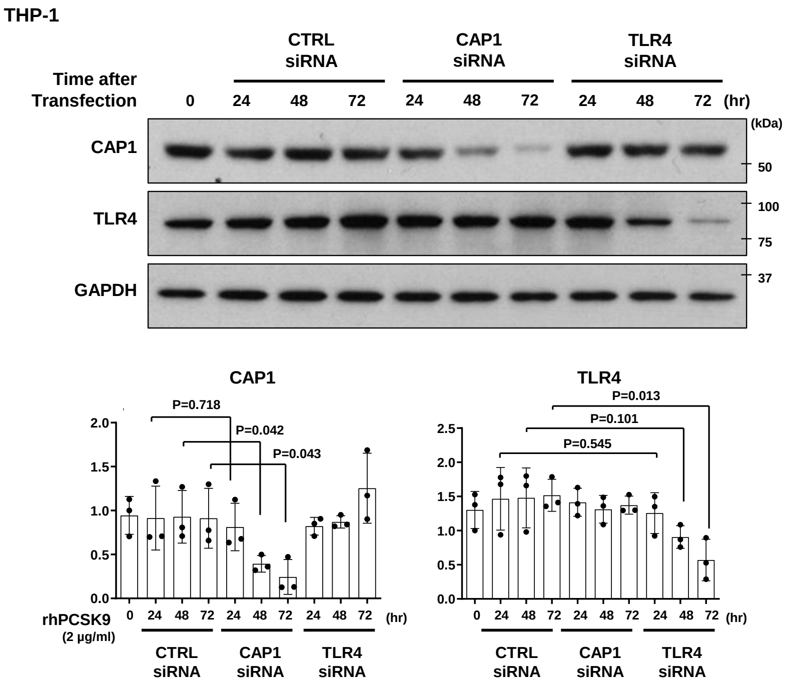

Supplementary Fig. 4

Immunoblot analysis demonstrating the effects of time elapsed after siRNA transfection on the degree of CAP1 or TLR4 knockdown (top). THP-1 cells were transfected with CAP1 or TLR4 siRNA and harvested at different time points: 0, 24, 48, and 72 hr (N=3). The representative figures of three independent experiments are shown and quantified using ImageJ software (bottom). The differences between the groups were compared using the unpaired t-test (two-tailed). All data are presented as mean values +/- SD.
