# Supplementary material for: PCSK9 stimulates Syk, PKCδ, and NF-κB, leading to atherosclerosis progression independently of LDL receptor
Source: Nat Commun. 2024 Mar 30;15:2789. doi: 10.1038/s41467-024-46336-2 (PMC10981688; doi:10.1038/s41467-024-46336-2)
Supplement: Supplementary file 3 — Reporting Summary [file 41467_2024_46336_MOESM3_ESM.pdf]

nature portfolio

Corresponding author(s): Hyun-Duk Jang, Hyo-Soo Kim

Last updated by author(s): November 14, 2023

Reporting Summary

Nature Portfolio wishes to improve the reproducibility of the work that we publish. This form provides structure for consistency and transparency in reporting. For further information on Nature Portfolio policies, see our Editorial Policies and the Editorial Policy Checklist.

Please do not complete any field with "not applicable" or n/a. Refer to the help text for what text to use if an item is not relevant to your study.
For final submission: please carefully check your responses for accuracy; you will not be able to make changes later.

Statistics

For all statistical analyses, confirm that the following items are present in the figure legend, table legend, main text, or Methods section.

n/a	Confirmed	
☐	■	The exact sample size (n) for each experimental group/condition, given as a discrete number and unit of measurement
☐	■	A statement on whether measurements were taken from distinct samples or whether the same sample was measured repeatedly
☐	■	The statistical test(s) used AND whether they are one- or two-sided<br>*Only common tests should be described solely by name; describe more complex techniques in the Methods section.*
■	☐	A description of all covariates tested
■	☐	A description of any assumptions or corrections, such as tests of normality and adjustment for multiple comparisons
☐	■	A full description of the statistical parameters including central tendency (e.g. means) or other basic estimates (e.g. regression coefficient) AND variation (e.g. standard deviation) or associated estimates of uncertainty (e.g. confidence intervals)
☐	■	For null hypothesis testing, the test statistic (e.g. F, t, r) with confidence intervals, effect sizes, degrees of freedom and P value noted<br>*Give P values as exact values whenever suitable.*
■	☐	For Bayesian analysis, information on the choice of priors and Markov chain Monte Carlo settings
■	☐	For hierarchical and complex designs, identification of the appropriate level for tests and full reporting of outcomes
☐	■	Estimates of effect sizes (e.g. Cohen's d, Pearson's r), indicating how they were calculated

Our web collection on statistics for biologists contains articles on many of the points above.

Software and code

Policy information about availability of computer code

Data collection	No specific software designed to collect data.
Data analysis	FlowJo (v10.0.5), ImageJ (plug ITCN) software (National Institutes of Health), GraphPad Prism (v8.4.3), SAINTexpress (https://saint-apms.sourceforge.net/Main.html), MaxQuant software (v1.6.1.0), and DAVID (v6.8) Amersham Imager 600, Zeiss LSM 710 confocal microscope, Leica STELLARIS 8, ABI Prism 7500 Sequence Detection System, QuantStudio™ 5 Real-Time PCR System

For manuscripts utilizing custom algorithms or software that are central to the research but not yet described in published literature, software must be made available to editors and r eviewers. We strongly encourage code deposition in a community repository (e.g. GitHub). See the Nature Portfolio guidelines for submitting code & software for further information.

Data

Policy information about availability of data

All manuscripts must include a data availability statement. This statement should provide the following information, where applicable:

- Accession codes, unique identifiers, or web links for publicly available datasets
- A description of any restrictions on data availability
- For clinical datasets or third party data, please ensure that the statement adheres to our policy

All data, except MASS data, are provided in this paper. Restricted access to the MASS data is due to our ongoing research on the functions and biology of the proteins binding to CAP1. The data supporting the findings of this study can be accessed from the corresponding authors upon a reasonable request.

Research involving human participants, their data, or biological material

Policy information about studies with human participants or human data. See also policy information about sex, gender (identity/presentation), and sexual orientation and race, ethnicity and racism.

Reporting on sex and gender	Sex and gender were neither considered nor indicated in this study
Reporting on race, ethnicity, or other socially relevant groupings	Race, ethnicity, or other socially relevant groupings were not considered in this study
Population characteristics	CAD patients were under prescribed statin medication, while healthy volunteers had no medications
Recruitment	We created a handout for patients with coronary artery disease (CAD) who came in for medical consultations and distributed it. We recruited participants from those who voluntarily chose to participate in the study. For healthy participants, we recruited individuals through posters and the official IRB promotion channels of Seoul National University Hospital. Potential participants were provided with detailed information about the study and its objectives. Informed consent was obtained from all participants, and ethical guidelines were followed throughout the recruitment process. Sampling bias of age may be present as CAD is the disease that is strongly related to aging. However, the age range of participants were from 30-60 in both CAD patients and healthy participants, which would reduce impact on our results, if there was any.
Ethics oversight	This study was approved by the Institutional Review Board of Seoul National University Hospital (IRB no. H-2208-112-1351). This research was conducted in accordance with the Helsinki Declaration.

Note that full information on the approval of the study protocol must also be provided in the manuscript.

Field-specific reporting

Please select the one below that is the best fit for your research. If you are not sure, read the appropriate sections before making your selection.

- [x] Life sciences
- [ ] Behavioural & social sciences
- [ ] Ecological, evolutionary & environmental sciences

For a reference copy of the document with all sections, see nature.com/documents/nr-reporting-summary-flat.pdf

Life sciences study design

All studies must disclose on these points even when the disclosure is negative.

Sample size	We did not employ any statistical method to pre-determine sample sizes; however, our sample sizes were consistent with those previously reported in our published research (Jang et al, Eur Heart J. 2020;41(2):239-252. doi:10.1093/eurheartj/ehz566)
Data exclusions	In serum profile in CAD patients, data of patients number 14 was excluded due to the failure in reading (HITACHI)
Replication	All the reported experiments were reproducible. All in vitro experiments were conducted a minimum of three independent repeated experiments and were reproducible.
Randomization	For the in vivo experiments, mice were assigned to experimental groups through random allocation. For in vitro experiments, samples were randomly allocated into different experimental groups
Blinding	We utilized blinding, including data analyst blinding and applied instrumentation blinding, to ensure unbiased and reliable results

Behavioural & social sciences study design

All studies must disclose on these points even when the disclosure is negative.

Study description	
Research sample	
Sampling strategy	
Data collection Ti	
ming	
Data exclusions	
Non-participation	
Randomization	

Ecological, evolutionary & environmental sciences study design

All studies must disclose on these points even when the disclosure is negative.

Study description	
Research sample	
Sampling strategy	
Data collection	
Timing and spatial scale	
Data exclusions	
Reproducibility	
Randomization	
Blinding	

Did the study involve field work? [ ] Yes [ ] No

Field work, collection and transport

Field conditions	
Location	
Access & import/export	
Disturbance	

Reporting for specific materials, systems and methods

We require information from authors about some types of materials, experimental systems and methods used in many studies. Here, indicate whether each material, system or method listed is relevant to your study. If you are not sure if a list item applies to your research, read the appropriate section before selecting a response.

Materials & experimental systems

n/a	Involved in the study	
[ ]	[x]	Antibodies
[ ]	[x]	Eukaryotic cell lines
[x]	[ ]	Palaeontology and archaeology
[ ]	[x]	Animals and other organisms
[x]	[ ]	Clinical data
[x]	[ ]	Dual use research of concern
[x]	[ ]	Plants

Methods

n/a	Involved in the study	
[x]	[ ]	ChIP-seq
[ ]	[x]	Flow cytometry
[x]	[ ]	MRI-based neuroimaging

Antibodies

Antibodies used

The primary antibodies used in this study were as follows: anti-NF-κB p65 (Santa Cruz Biotechnology, Santa Cruz, CA, USA; sc-109; WB 1:1000), anti-human CAP1 (Santa Cruz Biotechnology; sc-100917 WB 1:2000, IF 1:100), anti-human/mouse CAP1 (Santa Cruz Biotechnology; sc-134637; WB 1:2000 IF 1:100), anti-ICAM-1 (Santa Cruz Biotechnology; sc-8439; WB 1:1000), anti-ICAM-1 (Santa Cruz Biotechnology; sc-7891; WB 1:1000), anti-GAPDH (Sigma-Aldrich, St. Louis, MO, USA; G9545; WB 1:20,000), anti-VCAM-1 (Santa Cruz Biotechnology; sc-1504; WB 1:1000), anti-PCSK9 (Cell Signaling Technology, Danvers, MA, USA; #85813; WB 1:1000, IF 1:50), anti-p-SYK (Cell Signaling Technology; #2701S; WB:1000, IF 1:50), anti-p-PKCδ (Cell Signaling Technology; #2055S; WB 1:1000, IF 1:50, Abcam, Cambridge, UK; #109539; WB 1:1000), anti-p-AKT (Cell Signaling Technology; #4060S; WB 1:1000), anti-SYK (Cell Signaling Technology; #13198S; WB 1:1000), anti-PKCδ (Cell Signaling Technology; #9616S; WB 1:1000), anti-AKT (Cell Signaling Technology; #2920S; WB 1:1000), anti-F4/80 (Cell Signaling Technology; #30325; IF 1:100), and anti-αSMA (Sigma-Aldrich, St. Louis, MO, USA; #A2547; IF 1:200). For the secondary antibody, anti-mouse IgG horseradish peroxidase (HRP; Thermo Fisher Scientific [formerly called Invitrogen], Waltham, MA, USA; #31430; WB 1:5000), anti-goat IgG HRP (Invitrogen; #31403; WB 1:3000), or anti-rabbit IgG HRP (Invitrogen; #32460; WB 1:5000), Donkey anti-Mouse IgG Antibody, Alexa Fluor™ 488 (Invitrogen; A21202; IF 1:200~ 500), Donkey anti-Mouse IgG Antibody, Alexa Fluor™ 555 (Invitrogen; A31570; IF 1:200~ 500), Donkey anti-Rabbit IgG Antibody, Alexa Fluor™ 488 (Invitrogen; A21206; IF 1:200~ 500), Donkey anti-Rabbit IgG Antibody, Alexa Fluor™ 555 (Invitrogen; A31572; IF 1:200~ 500), Donkey anti-Goat IgG Antibody, Alexa Fluor™ 633 (Invitrogen; A21082; IF 1:200~ 500) was used.

Validation

The antibodies were purchased from Santa Cruz Biotechnology, Sigma-Aldrich, Cell Signaling Technology, and Thermo Fisher Scientific. All antibodies have been validated by each respective company through immunoblotting and immunostaining on various cell lines. Validation statements for all antibodies listed above can be found through the manufacturer's website. In experiments using cell lines and in vitro experiments using human PBMC-derived macrophages, the primary antibodies, which were validated for human species, were used. For experiments using mouse PBMCs and tissue staining experiments, the primary antibodies, which were validated for mouse species, were used

Eukaryotic cell lines

Policy information about cell lines and Sex and Gender in Research

Cell line source(s)	THP-1 (TIB-202) and HEK293T (CRL-3216) cells were obtained from ATCC (American Type Culture Collection), while HUVEC (C2519A) cells were sourced from Lonza
Authentication	STR profiling was conducted and authenticated by ATCC and 15 population doublings was guaranteed by Lonza
Mycoplasma contamination	Not detected
Commonly misidentified lines (See ICLAC register)	No commonly misidentified cell lines were used in the study.

Palaeontology and Archaeology

Specimen provenance	
Specimen deposition	
Dating methods	

☐ Tick this box to confirm that the raw and calibrated dates are available in the paper or in Supplementary Information.

Ethics oversight	

Note that full information on the approval of the study protocol must also be provided in the manuscript.

Animals and other research organisms

Policy information about studies involving animals; ARRIVE guidelines recommended for reporting animal research, and Sex and Gender in Research

Laboratory animals	Mice were housed in a specific-pathogen-free (SPF) facility with controlled environmental conditions, including a temperature range of 20-26°C, humidity maintained between 30-70%, and a standard 12-hour light/dark cycle. They were provided with ad libitum access to standard rodent chow and clean water, and cages were equipped with appropriate bedding, nesting material, and environmental enrichment items. Regular cage cleaning and sanitation were performed to ensure a hygienic environment, and noise levels were minimized to reduce stress on the animals. Age-matched (8-week-old) *Cap1*$^{+/+}$ mice, their *Cap1*$^{+/-}$ littermates, and age-matched (8-week-old) *Ldlr*$^{-/-}$ male mice were used for the carotid ligation model. All animals were housed in the IACUC accredited animal facility at Seoul National University Hospital, where the dark/light cycle, ambient temperature, and humidity were centrally-regulated.
Wild animals	This study did not involve wild animals
Reporting on sex	male mice were chosen to eliminate the influence of sex hormones and to take advantage of their greater propensity for developing atherosclerotic lesions in the mouse model
Field-collected samples	This study did not involve samples collected from the field.
Ethics oversight	All animal experiments were performed with approval from the Institutional Animal Care and Use Committee (IACUC, 17-0181-C1A0) of the Clinical Research Institute of Seoul National University Hospital, Republic of Korea

Note that full information on the approval of the study protocol must also be provided in the manuscript.

Clinical data

Policy information about clinical studies

All manuscripts should comply with the ICMJE guidelines for publication of clinical research and a completed CONSORT checklist must be included with all submissions.

Clinical trial registration	
Study protocol	
Data collection	
Outcomes	

Dual use research of concern

Policy information about dual use research of concern

Hazards

Could the accidental, deliberate or reckless misuse of agents or technologies generated in the work, or the application of information presented i n the manuscript, pose a threat to:

No	Yes	
☐	☐	Public health Na
☐	☐	tional security
☐	☐	Crops and/or livestock
☐	☐	Ecosystems
☐	☐	Any other significant area

Experiments of concern

Does the work involve any of these experiments of concern:

No	Yes	
☐	☐	Demonstrate how to render a vaccine ineffective
☐	☐	Confer resistance to therapeutically useful antibiotics or antiviral agents
☐	☐	Enhance the virulence of a pathogen or render a nonpathogen virulent
☐	☐	Increase transmissibility of a pathogen
☐	☐	Alter the host range of a pathogen
☐	☐	Enable evasion of diagnostic/detection modalities Ena
☐	☐	ble the weaponization of a biological agent or toxin
☐	☐	Any other potentially harmful combination of experiments and agents

Plants

Seed stocks

Novel plant genotypes

Authentication

ChIP-seq

Data deposition

☐ Confirm that both raw and final processed data have been deposited in a public database such as GEO.

☐ Confirm that you have deposited or provided access to graph files (e.g. BED files) for the called peaks.

Data access links
May remain private before publication.

Files in database submission

Genome browser session
(e.g. UCSC)

Methodology

Replicates

Sequencing depth

Antibodies

Peak calling parameters

Data quality

Software

Flow Cytometry

Plots

Confirm that:

- [x] The axis labels state the marker and fluorochrome used (e.g. CD4-FITC).
- [x] The axis scales are clearly visible. Include numbers along axes only for bottom left plot of group (a 'group' is an analysis of identical markers).
- [ ] All plots are contour plots with outliers or pseudocolor plots.
- [x] A numerical value for number of cells or percentage (with statistics) is provided.

Methodology

Sample preparation

For surface FACS analysis, THP-1 cells were incubated with recombinant human PCSK9 (500, 1000, 2,000 ng/ml) for 1 hour. After incubation, the cells were fixed with 1% formaldehyde and washed with cold phosphate-buffered saline (PBS). Following centrifugation, the cells were stained with anti-human CAP1 antibody (Santa Cruz Biotechnology; sc-134637) and anti-rabbit IgG Alexa Fluor 488 (Invitrogen; A-21206) secondary antibodies.
To assess ox-LDL uptake, THP-1 cells were treated with CTRL or CAP1 siRNA and pre-incubated with recombinant human PCSK9 (2 μg/ml) for 30 minutes. Subsequently, 10 μg/ml of DiI-ox-LDL (Thermo Fisher Scientific; L34358) was added for 1 hour, and the cells were then fixed with 1% formaldehyde and washed with cold PBS.
To assess VLA-4 activation, THP-1 cells were transfected with CTRL or CAP1 siRNA and left untreated or were treated with rhPCSK9 (2 μg/ml). After centrifugation, the cells were washed with FACS buffer and probed with several antibodies specific for VLA-4 (Merck Millipore, Burlington, MA, USA; FCMAB389F).

Instrument

FACSCanto Ⅱ (BD Biosciences).

Software

BD FACSDiva software

Cell population abundance

Established a positive gate by setting the isotype control as negative

Gating strategy

Based on forward and side scatter to distinguish populations, positive gating was obtained using single parameter histograms, with isotype control set as negative

- [x] Tick this box to confirm that a figure exemplifying the gating strategy is provided in the Supplementary Information.

Magnetic resonance imaging

Experimental design

Design type

Design specifications

Behavioral performance measures

Imaging type(s)

Field strength

Sequence & imaging parameters

Area of acquisition

Diffusion MRI ☐ Used ☐ Not used

Preprocessing

Preprocessing software

Normalization Normalizati

on template Noise and art

ifact removal Volume cens

oring

Statistical modeling & inference

Model type and settings

Effect(s) tested

Specify type of analysis: ☐ Whole brain ☐ ROI-based ☐ Both

Statistic type for inference

(See Eklund et al. 2016)

Correction

Models & analysis

n/a	Involved in the study
☐	☐ Functional and/or effective connectivity
☐	☐ Graph analysis
☐	☐ Multivariate modeling or predictive analysis

Functional and/or effective connectivity

Graph analysis

Multivariate modeling and predictive analysis

This checklist template is licensed under a Creative Commons Attribution 4.0 International License, which permits use, sharing, adaptation, distribution and reproduction in any medium or format, as long as you give appropriate credit to the original author(s) and the source, provide a link to the Creative Commons license, and indicate if changes were made. The images or other third party material in this article are included in the article's Creative Commons license, unless indicated otherwise in a credit line to the material. If material is not included in the article's Creative Commons license and your intended use is not permitted by statutory regulation or exceeds the permitted use, you will need to obtain permission directly from the copyright holder. To view a copy of this license, visit http://creativecommons.org/licenses/by/4.0/
